# An integrated model of gene-culture coevolution of language mediated by phenotypic plasticity

**DOI:** 10.1038/s41598-018-26233-7

**Published:** 2018-05-23

**Authors:** Tsubasa Azumagakito, Reiji Suzuki, Takaya Arita

**Affiliations:** 1 Graduate School of Information Science, Nagoya University, Furo-cho Nagoya, 464-8601 Japan; 2 Graduate School of Informatics, Nagoya University, Furo-cho Nagoya, 464-8601 Japan

## Abstract

In this paper, we propose an agent-based model for investigating possible scenarios of genetic and cultural language evolution based on an integrated gene-culture coevolutionary framework. We focused on the following problems: (1) how communicative ability can evolve directionally under positive frequency-dependent selection and (2) how much of the directional effect there is between language and biological evolution. In our evolutionary experiments and analysis, we discovered a coevolutionary scenario involving the biological evolution of phenotypic plasticity and a cyclic coevolutionary dynamic between genetic and cultural evolution that is mediated by phenotypic plasticity. Furthermore, we discovered that the rates of cultural change are usually faster than the biological rates and fluctuate on a short time scale; on a long time scale, however, cultural rates tend to be slow. This implies that biological evolution can maintain the pace with language evolution. Finally, we analyzed the transfer entropy for a quantitative discussion of the directional effects between both evolutions. The results showed that biological evolution appears to be unable to maintain the pace with language evolution on short time scales, while their mutual directional effects are in the same range on long time scales. This implies that language and the relevant biology could coevolve.

## Introduction

The possession of language distinguishes humans from other animals. It is true that other animals also engage in vocal communication; for example, vervet monkeys can convey some simple information by using alert calls^1^. However, the vocal communication of such animals lacks the complex grammar and high expressiveness that characterize human language. Why do only humans have sophisticated language? This is one of the core questions to understanding human identity. In this study, we explored the evolution of language in the context of the biological evolution of the fundamental traits underlying communicative interaction. We focused on two fundamental problems concerning language evolution: how communicative ability can evolve directionally under frequency-dependent selection and whether the cultural evolution of language and biological evolution of traits underlying communicative interactions can coevolve.

One of the fundamental assumptions of most studies on language evolution from the biological viewpoint is that the fundamental traits underlying communicative interactions evolved under directional selection. These traits can be modified incrementally to increase the benefit from communicative interactions. At the same time, it has been assumed that such traits must be shared between individuals for communication to succeed. Accordingly, at least some of the selection is positively frequency-dependent. This may obstruct evolution based on directional selection. We believe that this captures a fundamental and general problem in the evolution of communicative traits. For example, in the context of language evolution, it has been pointed out that mutations in grammar cannot be beneficial because the peers of an individual with a grammar mutation may not understand the mutant form^2^.

Nature’s solution to this challenging problem can be found in the evolution of phenotypic plasticity^3^. Phenotypic plasticity refers to the variability in a phenotype obtained from a given genotype resulting from development in different environments^4^. In the field of evolutionary biology, ontogenetic adaptation (individual learning) based on phenotypic plasticity has recently been recognized as one of the key factors that bring about the adaptive evolution of novel traits^5,6^. Wund summarized eight hypotheses on how plasticity may influence evolution (including several pieces of empirical support), focusing mainly on the adaptation to new environments^7^. For example, it has been suggested that phenotypic plasticity promotes persistence in a new environment and that a change in the environment can release cryptic genetic variations via phenotypic plasticity which in turn impact the rate of the evolutionary response. Zollman and Smead^8^ analyzed simple models of language evolution based on Lewis’s signaling game and the prisoner’s dilemma game. They observed that the presence of plastic individuals alters the trajectory of evolution by directing the population away from non-adaptive signalling and toward optimal signalling. They termed this the “Baldwin optimizing effect.” Suzuki and Arita showed that such an adaptive shift can occur repeatedly by using a computational model of the evolution of communicative traits (e.g., signaling and receiving behaviors^9,10^, channels^11^) that incorporate behavioral plasticity. These studies have indicated that learning may be an important driving force for adaptive evolution in the context of communicative interactions, although they did not explicitly deal with the cultural evolution of language.

The second problem that we considered is the relationships between two different evolutionary processes: biological evolution and cultural evolution^12^. Evolutionary scholars have converged on the idea that the cultural and innate aspects of language were tightly linked in a process of gene-culture coevolution^13^. However, the relationship between genes and language is extremely complex and shrouded in controversy because these mechanisms interact with each other despite the difference in their time scales^14^. Substantial knowledge regarding gene–culture coevolution in general has been acquired from the viewpoint of genetic analysis^15^. One seemingly common argument is that language changes rapidly and is a “moving target;” therefore, it does not provide a stable environment for biological adaptations^16^. This argument is almost parallel with the assumption that biological evolution has become “frozen” as if language evolution works on a “fixed” biological background^17^. For example, Chater *et al*.^18^ used a computational model to show that there are strong restrictions on the conditions under which the Baldwin effect (typically interpreted as a two-step evolution of the genetic acquisition of a learned trait without the Lamarckian mechanism^19^) can embed arbitrary linguistic constraints and that the effect emerges only when language provides a stable target for natural selection.

However, problems have been posed for the underlying assumptions of the “moving target argument.” With regard to biological evolution, Számadó *et al*.^20^ summarized several ways in which natural selection can adapt to moving targets. The simplest way is genetic evolution. In general, the ability of a population to keep pace with change depends on both the size of the population and the variation present. There are indeed many examples showing that adaptation can be very fast if variability is present. Furthermore, it has been reported that the rate of genomic evolution during the last 40,000 years has been more than 100 times higher than the characteristic rate for most of human evolution^21^. The second way is again the phenotypic plasticity. When natural selection acts to preserve adaptive phenotypes, it can lead to genetic change and to the fixation of the preserved adaptive phenotype by several evolutionary processes, including the Baldwin effect^22^. The third way is by means of systems and organs that have evolved to cope with fast-changing environments, much like the immune system being capable of tracking most pathogens.

With regard to cultural evolution, Számadó *et al*.^20^ summarized two possible reasons why cultural evolution may not present a moving target that cannot be tracked by biological evolution; this is partly due to the fact that the rate of linguistic change depends on the frequency of use and population size. First, even contemporary linguistic changes need not be that fast. Second, past rates of linguistic change may have been much slower. There is also a seemingly counterintuitive claim^23^ that a “moving target” should increase the rate of evolution because temporally varying goals have been shown to substantially accelerate evolution compared with evolution under a fixed goal.

Another factor is the measurement of the evolutionary rates. In general, there is an inverse correlation between the rates of evolution and the time interval used to measure the rates. This can explain why the pace of cultural evolution appears faster, that is usually measured with shorter intervals^24^. We discuss this factor in detail later in the section Evolutionary Rates.

**Figure 1:**
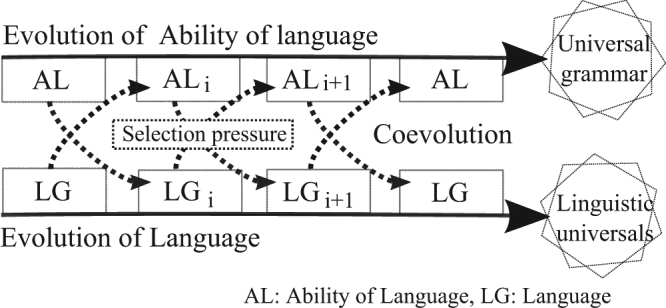
Coevolution between the language and language ability. The upper arrow represents the evolution of language ability in general. The lower arrow represents the evolution of language itself. The language ability and language are represented by *AL* and *LG*, respectively. *AL*_*i*_ controls the selection pressures shaping *LG*_*i*+1_; conversely, *LG*_*i*_ controls the selection pressures shaping *AL*_*i*+1_. Universal grammar (the theory proposing that the ability to learn linguistic grammar is hard-coded into the brain) and linguistic universals (general patterns that potentially exist in almost all natural languages) might have emerged as inevitable results of this coevolution.

Rather than viewing language as a monolithic and independent entity, modern researchers typically break it down into its component mechanisms and analyze these independently^25^. These studies tend to be one-sided. Indeed, there has been relatively little work on investigating the two types of adaptations within a single framework^26^. Based on the above considerations and hypotheses, in this study we used a coevolutionary framework that allows us to integrate biological and cultural evolution to develop a comprehensive understanding of language evolution. Figure 1 illustrates a general picture of the coevolution, in which there are two intertwined adaptation processes: language adapts to the brain, and the brain adapts to language. On the one hand, a language is continuously changed by its users, which brings about linguistic variations much like mutation brings about genetic variation. Language variants that have more fitness contribution to their users in terms of, for example, learnability and expressiveness tend to survive and spread in the population of languages. On the other hand, having innate linguistic abilities that equip an individual to handle more sophisticated language variants better than others provides a fitness advantage. In addition to biological evolution and cultural evolution, we considered individual learning based on phenotypic plasticity as a third adaptive system, which was assumed to play a key role in considering the two fundamental problems that were the focus of this study.

We present a minimum computational model with a one-dimensional linguistic space, in which biological evolution, cultural evolution and individual learning can evolve language. It is a sophistication of our previous models with a two-dimensional linguistic space^27–29^. This sophistication could realize our quantitative analysis on the model using evolutionary rates and transfer entropy in this paper.

## Models

We propose an integrated computational framework for investigating possible scenarios of the genetic and cultural evolution of language. This framework allowed us to study coevolutionary interactions between languages and agents who use the languages for communication.

### Language and Agent

**Figure 2:**
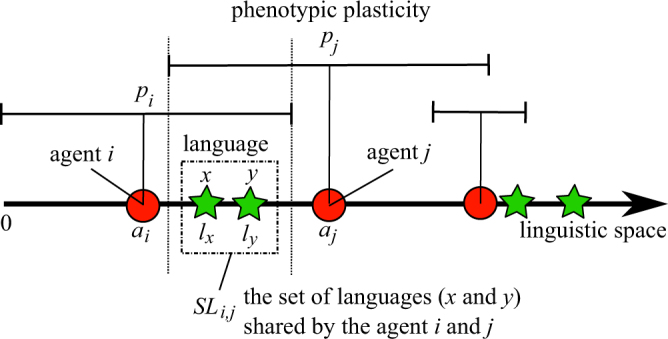
The linguistic space.

There are a finite number of agents and languages in a one-dimensional space, and agents can communicate with each other by using their shared languages (Fig. 2). Note that the number of agents is *N* and does not change through a trial, while the number of languages can vary.

Each language is defined as a point in the space. The position *l*_*x*_ (≥0) of a language *x* in the space represents the expressiveness of the language, which is the expected fitness benefit of a successful communication using that language.

Each agent *i* is also represented as a point in the same space and the interval surrounding the point. This point represents the agent’s innate language ability, for which the position is determined by its genotype *a*_*i*_ (≥0). *a*_*i*_ also represents a possible language that can be learned by the agent *i* with the minimum innate cost. In other words, the agent can use the corresponding language located at *a*_*i*_ in the linguistic space without learning if it exists in the current population of languages. The area represents the agent’s linguistic plasticity determined by its genotype *p*_*i*_. The agent can use any languages that exist in its plasticity interval [*a*_*i*_ − *p*_*i*_, *a*_*i*_ + *p*_*i*_] through the learning processes of the languages by paying a certain cost (explained below in detail).

### Linguistic Interactions

In each generation, there is a chance for communication between two agents in each of all possible pairs of agents. If there are one or more languages that can be used by both agents, the agents can communicate successfully by using one of their shared languages. Specifically, the set of shared languages *SL*_*i*,*j*_ between agents *i* and *j* is defined as follows:

1 $$S{L}_{i,j}=\{x|({a}_{i}-{p}_{i}\le {l}_{x}\le {a}_{i}+{p}_{i})\cap ({a}_{j}-{p}_{j}\le {l}_{x}\le {a}_{j}+{p}_{j})\}.$$

The agents can communicate successfully if *SL*_*i*,*j*_ ≠ /0.

The fitness of each agent is determined by summing the expressiveness of the languages used in its successful communication with others and the cost of the linguistic plasticity. The fitness of an agent *i* is defined as follows:

2 $$fitnes{s}_{i}={W}_{1}\cdot \sum _{j\,\in \,S{C}_{i}}{l}_{i,j}-{W}_{2}\cdot {({p}_{i}+1)}^{{a}_{i}},$$

where *W*_1_ and *W*_2_ are the weights for the two components of the fitness function. The first component represents the benefit from successful communicative interactions. *SC*_*i*_ is the set of agents with which the focal agent *i* successfully communicates by using the shared language with the position *l*_*i*,*j*_. Note that, if the communicating agents share two or more languages in their communication, one of the shared languages is randomly selected and used for calculating the fitness. The second component represents the cost of linguistic plasticity. It is determined by the size of the interval of its plasticity (*p*_*i*_). The cost increases exponentially as the agents’ innate ability increases. This reflects a situation in which a greater innate ability makes it more costly to maintain the learning ability (e.g., the cost for maintaining the larger brain size). Overall, this definition of the fitness means that agents who can communicate with more other agents by using more expressive languages that are acquired with less linguistic plasticity will have higher fitness.

### Biological Evolution of Language Ability

After communicative interactions among agents, the biological evolution of agents occurs. The population of the next generation is generated by repeating the following procedures *N* times: a parent agent for the next generation is selected by using a roulette wheel selection that is proportional to the fitness (i.e., the probability that an agent is picked up as a parent is proportional to its fitness), which produces an offspring that has the same genotypes as the ones of its parent. Each genotype of the offspring is mutated with the probability *P*_*m*_. A mutation process adds a small random value R(0, 2) to the original genetic value of an offspring *i* (*a*_*i*_ and *p*_*i*_), where R(*μ*, *σ*^2^) is a normal random number with the mean *μ* and variance *σ*^2^.

### Cultural Evolution of Language

Subsequently, the population of languages evolves according to the following four cultural processes: cultural change, division, extinction, and fusion.

#### Cultural change

**Figure 3:**
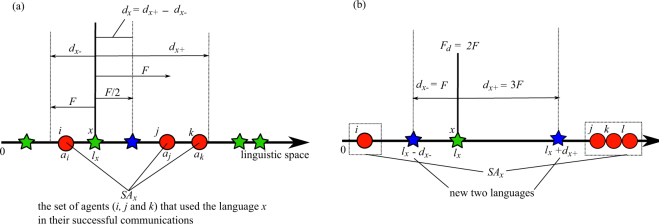
(**a**) Example of a step in cultural language evolution where *n*_*i*_ = 1, *n*_*j*_ = 2, and *n*_*k*_ = 1. (**b**) Example of a language division process where *n*_*i*_ = 1, *n*_*j*_ = 1, *n*_*k*_ = 1, and *n*_*l*_ = 1.

We define a cultural change in languages as a change in the position of the language in the linguistic space due to the use of the language during successful communication among agents. Basically, a successful communication between a pair of agents moves the language used in the communication toward the agents’ innate linguistic abilities, as shown in Fig. 3(a).

Specifically, the direction and amount of displacement (*d*_*x*_) of a language *x* is calculated as follows:

3 $${d}_{x}={d}_{x+}+{d}_{x-},$$

4 $${d}_{x+}=\{{\sum }_{i\in s{a}_{x}}\begin{array}{cc}F/{n}_{i} & \,{\rm{if}}\,{a}_{i} > {l}_{x},\\ 0 & \,{\rm{otherwise}},\end{array}$$

5 $${d}_{x-}=\{\sum _{i\,\in \,s{a}_{x}}\begin{array}{cc}F/{n}_{i} & {\rm{if}}\,{a}_{i} < {l}_{x}\,,\\ 0 & \,{\rm{otherwise}}{\rm{.}}\,\end{array}$$

*d*_*x* +_ and *d*_*x*−_ are the total amounts of displacement toward the positive and negative directions, respectively. *SA*_*x*_ is the set of agents that used the language *x* in their successful communications at least one time. *n*_*i*_ is the number of languages that the agent *i* is able to use, i.e., languages that are in the plasticity interval of the agent *i*. *F* is the parameter that determines the amount of displacement. Each language *x* moves by *d*_*x*_ in the linguistic space.

#### Division

Each language is divided into two languages if it is pulled dramatically toward opposite directions simultaneously. Specifically, the language *x* is divided when min(*d*_*x*+_, *d*_*x*−_) > *F*_*d*_. Instead of the focal language *x* being removed, new two languages are created and placed at *l*_*x*_ + *d*_*x*+_ and *l*_*x*_ + *d*_*x*−_ in the linguistic space.

#### Extinction

Any languages that are not used by any agents in the current generation will not appear in the next generation; in other words, they are removed from the linguistic space. This represents language extinction.

#### Fusion

When the distance between two languages is fairly close, these languages are united into a single language. This process occurs when the difference between the two languages is smaller than the threshold *T*_*f*_. The united language is placed in the midpoint between these two languages.

Through the above processes, the populations of the agents and languages coevolve.

### One dimensional expression of language

Language is a communication tool but also a cognitive tool. Indeed, in the brain, utilizing language-related circuits, some form of linguistic knowledge is linked to the external world by producing/perceiving sounds and gestures, and at the same time, is connected to the inner mental world composed of concepts, intentions and reasoning^30^. We can also assume that, in general, the collective adaptivity of language is related with not only communicative but also cognitive aspects. If so, the one dimensional space in the model might include the cognitive aspects of language (e.g. recursive ability or ability to merge), although we focus on the communicative aspect of language and define the dimension as expressiveness in this paper.

In our previous study^29^, we constructed a two-dimensional model based on a polar coordinate system in which the distance from a language to the origin represents its expressiveness, and its angle represents its structural character. The similarity in the structural character determines a communication success between agents but does not affect the fitness value from that successful communication. We observed a “linguistic burst” in that many languages with structural differences emerged from successful communication among agents sharing a few languages in the initial population located at the origin. However, after a few hundred generations, the structural properties of languages converged to a certain value and a cyclic coevolutionary process began to occur, which is similar to the one discussed in this paper. This paper proposed a more simplified model to focus more on evolutionary rates of biological and cultural processes and their directional effects, using transfer entropy.

## Results

We conducted evolutionary experiments for 10,000 generations. The following parameters were used: *N* = 2000, *W*_1_ = 3, *W*_2_ = 10, *P*_*m*_ = 0.001, *F*_*d*_ = 0.008, *T*_*f*_ = 0.02, and *F* = 0.001. The initial values of *a*_*i*_, *p*_*i*_, and *l*_*x*_ were randomly selected from [0, 1]. We selected these values so that division and fusion of languages continuously occurred. Especially, the effects of changing parameters *W*_2_, *F*, *F*_*d*_ and *T*_*f*_ are described later in this section.

**Figure 4:**
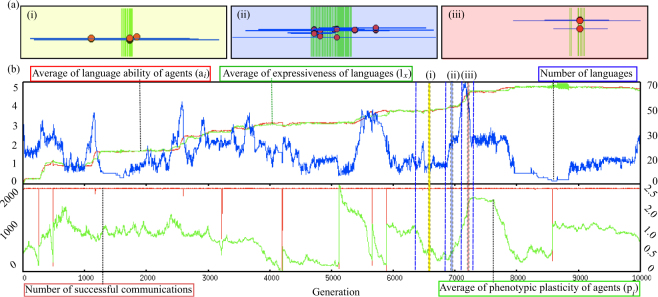
Example of the evolutionary process. (**a**) Several snapshots of the distribution of languages and agents in the linguistic space. The red circles represent the agents, and the blue line surrounding them represent their linguistic plasticity. The green lines express the languages. The x-axis represents language ability for agents (*a*_*i*_) and expressiveness of language (*l*_*x*_). The y-axis represents fitness of agents. (**b**) Evolution plots. The upper part shows the values of expressiveness of the language (green) and the agent’s innate language (red) during a typical evolution, which corresponds to the centers of their distributions in the linguistic space. The blue line represents number of languages. The lower part shows the average phenotypic plasticity of the agents (green) and the number of successful communications (red).

**Figure 5:**
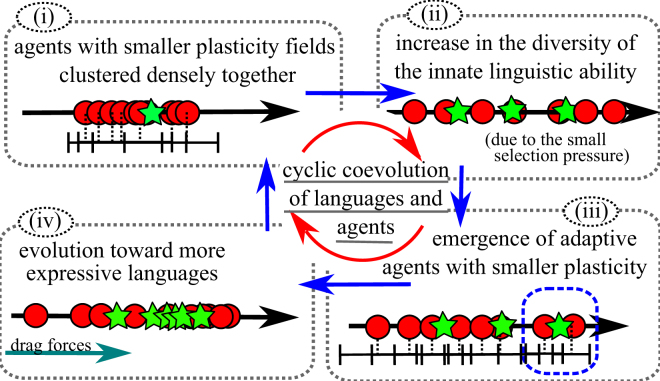
Typical scenario of the coevolutionary process.

Figure 4 shows an example run of this experiment. We observed the cyclic coevolutionary processes of languages and agents, which are summarized in Fig. 5. As an example, consider the evolutionary process from the 6500th to 7400th generations (i–iii). (i) Around the 6500th generation, we observed agents with smaller plasticity fields clustered densely together. In this situation, there was only weak selection pressure on the innate language ability because agents could already communicate successfully. (ii) This lack of selection pressure led innate language abilities to be scattered by neutral evolution around the 7000th generation. The number of languages increased during term (ii) because the increased diversity of the agents created more linguistic changes. (iii) Around the 7200th generation, some agents with more expressive innate language ability and lower phenotypic plasticity appeared and occupied the population quickly. Instead of communicating with many agents by using less expressive languages, these agents communicated with a limited number of neighbors by using more expressive languages while incurring only a small plasticity cost. This resulted in a net relative fitness gain. At the same time, the number of languages increased because the languages were dragged by two groups: the group of agents with a more expressive language ability and the group of the agents with a less expressive language ability. Afterwards, the language population evolved toward the languages used by the former group of adaptive agents via a process of cultural evolution arising from the increased use of the more expressive languages. Languages distant from the agents’ (shifted) innate language abilities became extinct, which led to a gradual decrease in the number of languages. As a result, the average expressiveness of language caught up with the innate language abilities, which means that the populations of agents and languages moved in an outward direction in the linguistic space, and their evolutionary process went back to the initial state of the cycle.

We conducted experiments to study the effects of the model’s parameters on the evolutionary process. First, to investigate the effect of the learning cost, we conducted experiments with various weights for the learning cost *W*_2_. We found that the duration until the population reached the coevolution phase increased with *W*_2_. A higher cost of learning placed the population under stronger selection pressure at low plasticity. Because individuals with low plasticity were less robust against mutations and often failed to leave offspring, the evolution speed dropped. The rate of increase in the expressiveness of languages was inversely proportional to *W*_2_ due to the increased duration until the start of the coevolutionary phase. For example, in the case of no cost (*W*_2_ = 0), the duration was quite short: the coevolutionary phase started after about 100 generations. In the case of a huge learning cost (*W*_2_ = 100000), the evolution of the language and population stagnated around the origin because individuals could not increase their plasticity at all. Note that higher values of *W*_2_ led to a shorter cycle period. This is thought to be because the rapid decrease in phenotypic plasticity (ii) tended to occur more often as the cost of plasticity increased.

We also investigated the effects of *F*, which determines the amount of displacement of languages. Because this parameter is used in the processes of cultural change and language division, we assumed the condition that the threshold for the division *F*_*d*_ is proportional to *F* (*F*_*d*_ = *F* × 300) in order to mainly focus on the effects of change in *F* on the cultural change process. Experiments with different settings of *F* (from 1.0 × 10^−7^ to 1.0 × 10^−4^) showed that the chances of all languages dying off during the early generations increased with *F*. This is because, at large *F* when there are many individuals changing language, the amount of displacement of languages tends to be so large that languages are displaced outside the plasticity range of the agent population. Especially in trials with high *F* (1.0 × 10^−5^), successful evolution was only observed when the initial population had high plasticity by chance. At extremely high *F* (1.0 × 10^−4^), all trials failed in about 10 generations.

Concerning *F*_*d*_ and *T*_*f*_, the thresholds for division and fusion of language, respectively, *F*_*d*_ is smaller, the less frequently division happens, and *T*_*f*_ is bigger, the more frequently fusion happens. In this case, the evolution goes stagnate because few languages can mediate communication between agents. Inversely, when *F*_*d*_ is too large, or *T*_*f*_ is too small, many languages tend to appear in one simulation step, leading to interesting behavior such as “linguistic burst”. We do not focus on this to simplify the following analyses in this paper.

### Analysis

#### Evolutionary Rates

**Figure 6:**
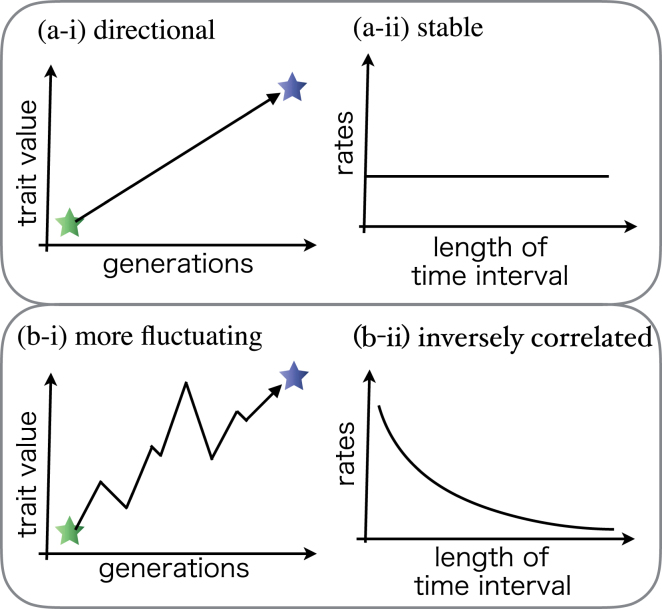
Examples of evolutionary trends for two quantitative traits and their associated evolutionary rates as a function of the measurement time interval.

Cultural linguistic change is often assumed to be significantly faster than biological change^18^. However, the rate of evolution is known to depend on the time interval over which the rates are measured^31^. Rates of evolution can be measured in *darwins* (d), which is a standardized unit of change in factors of *e*, the base of the natural logarithm, per million years^31^.

6 $$d=(\mathrm{ln}({v}_{2})-\,\mathrm{ln}({v}_{1}))/{\rm{\Delta }}t$$

where *v*_1_, *v*_2_, and Δ*t* are the mean trait value calculated at the time *t*_1_, the mean value calculated at the time *t*_2_, and the time interval between *v*_1_ and *v*_2_, respectively. Figure 6 illustrates an example of the measurement interval dependence of the evolutionary rate of a quantitative trait. If the evolutionary process is directional (a-i), then the rates are stable irrespective of the measurement time interval (a-ii). However, if the evolutionary process is less directional or fluctuating (b-i), the rates are inversely correlated with the measurement time interval (b-ii). This is because the fluctuation strongly affects the measured rate when the interval is short, while the general trend affects the rate when the interval is long. Perreault compared the rate of cultural and biological evolution by analyzing archaeological data. He found that the rates of cultural evolution are also inversely correlated with the measurement interval and concluded that cultural evolution is faster than biological evolution even when such a correlation is taken into account^24^. This has never been tested empirically before this study. We focused on this comparison in the context of language evolution, where the main challenge is the lack of empirical data. It is unclear as to what extent his findings can be extended to other domains of human cultures including language. Language evolution has at least two features which restrict the evolutionary rate: (1) Language evolution is restricted by the capacity of the human brain or organs that are related to language use, which differs from general cultural evolution. (2) Language speakers must share the conventions of language to communicate with each other, which restricts language evolution. Our model reflects these features. Thus, we were able to consider the relationship between both evolutionary processes in this context by performing informational analysis on our simulation results.

We measured the evolutionary rates of languages and agents to clarify the relationship between the rates of both evolutions by focusing on their measurement-interval dependence. Here, we calculated biological rates by using the agent’s mean trait values in the population in each generation with *a*_*i*_ of agents being used as the trait value. The cultural rates were calculated by focusing on each occurrence of the cultural process as follows. (a) Cultural change: *l*_*x*_ of the changed language was regarded as *v*_2_, and the corresponding value before its change was regarded as *v*_1_. (b) Division: in this case, two rates were calculated because one language was divided into two. The two different *l*_*x*_ values of the divided languages were regarded as *v*_2_, and *l*_*x*_ of the language before division was regarded as *v*_1_. (c) Fusion: this event also generated two rates because two languages fused into one. The *l*_*x*_ value of the fused language was regarded as *v*_2_, and the two different *l*_*x*_ values of the languages before fusion were regarded as *v*_1_. (d) Extinction: no rate was calculated. Here, we used the various lengths in generations of our model as Δ*t* in order to observe the effects of the measurement interval on the evolutionary rates of languages and agents.

**Figure 7:**
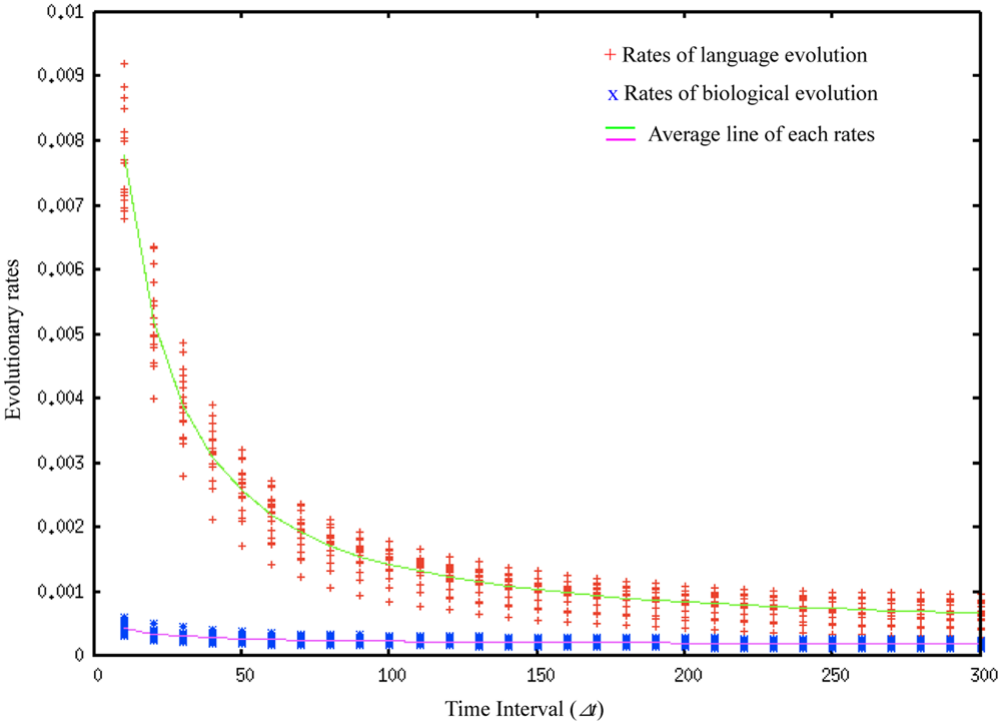
Evolutionary rates of language evolution and biological evolution.

Figure 7 shows the evolutionary rates of language and biological evolutions. We measured the rates in 18 experiments while changing Δ*t* for ten intervals. The x-axis represents the time interval (Δ*t*), and the y-axis represents the evolutionary rates of language and biological evolution (*darwins*).

The rates of language evolution tended to be higher than those of biological evolution. However, the rates of language evolution slowed with the time interval. This implies that the evolutionary rate of language has a stronger measurement-interval dependence than the rate of biological evolution. We propose that this is due to the lack of directionality in cultural evolution. This implies that biological evolution is more directional than cultural language evolution and can therefore maintain the pace with language evolution.

#### Transfer Entropy

We believe that it is important to investigate the directional effects for both evolutions in gene–culture coevolution to understand the complex relationship between them. In order to do this, we used transfer entropy (TE)^32^, which is able to quantify the asymmetric impact between multiple sequences. To put it simply, the TE from a process X to another process Y is the amount of uncertainty reduced in future values of Y by knowing the past values of X given past values of Y.

The TE *T*_*Y*→*X*_ from the sequence *Y*_*t*_ = {*y*_*t*_}_*t*=1,2,..._ to another sequence *X*_*t*_ = {*x*_*t*_}_*t*=1,2,..._ indicates the amount of uncertainty reduced in the state *x*_*t* + 1_ by knowing the past *l* states of ${y}_{t}^{l}=\{{y}_{t-l-1},\mathrm{...}\,{y}_{t}\}$ given the past *m* states of ${x}_{t}^{m}=\{{x}_{t-m-1},\mathrm{...}\,{x}_{t}\}$. *T*_*Y*→*X*_ is calculated as follows^32^:

7 $${T}_{Y\to X}=\sum _{{x}_{t+1},{x}_{t}^{m},{y}_{t}^{l}}p({x}_{t+1},{x}_{t}^{m},{y}_{t}^{l}){log}\,\frac{p({x}_{t+1}|{x}_{t}^{m},{y}_{t}^{l})}{p({x}_{t+1}|{x}_{t}^{m})}$$

This measure reflects the directional effect from the sequence *Y*_*t*_ to the sequence *X*_*t*_.

In this analysis, we generated discrete sequences from the continuous data in evolutionary experiments that consisted of the time series of average values of *l*_*x*_ and *a*_*i*_ in each generation. The sequence was generated as follows: We separated the time series of *l*_*x*_ (or *a*_*i*_) into periods with a specific time interval (Δ*t*) and generated a sequence composed of the average of *l*_*x*_ (or *a*_*i*_) in each separated period. Then, we replaced each value in the sequence with one of the five equally divided levels according to its rate of change from the previous value.

In addition, we calculated the effective transfer entropy (ET) to verify the significance of the effects of the calculated *T*_*Y*→*X*_^33^ by comparing it to the one when *Y*_*t*_ was randomized (${T}_{{Y}_{rand}\to X}$). *Y*_*rand*_ was generated by randomly shuffling the order of values in the discretized sequence of *Y*_*t*_. We created *Y*_*t*_ values by using different random seeds and calculated the mean values of ${T}_{{Y}_{rand}\to X}$ s, which can be considered as TE when *Y*_*t*_ has no relation with *X*_*t*_. We defined the ET (*ET*_*Y*→*X*_) as follows:

8 $$E{T}_{Y\to X}={T}_{Y\to X}-mean({T}_{{Y}_{rand}\to X})$$

**Figure 8:**
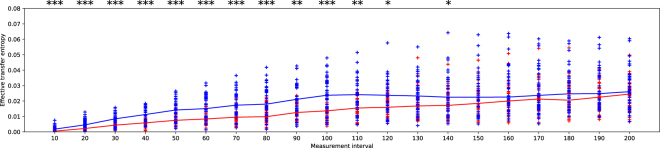
Effective transfer entropies for each trial against the time interval.

Figure 8 shows the ET against the time interval. We used *m* = *l* = 1. We measured the entropies of 38 experiments while changing Δ*t* for 10 intervals. The entropies were plotted as points for each trial. The X-axis represents the time interval (Δ*t*) used for generating sequences, and the Y-axis represents the effective transfer entropy with the corresponding Δ*t*. Each red point represents the effective entropy from language to biological evolution (*ET*_*L*→*B*_), and each blue point represents the ET from biological evolution to language evolution (*ET*_*B*→*L*_). The lines show their median values with the corresponding setting of (Δ*t*). The mark above each interval indicates the significance level of the difference between the median values of *ET*_*L*→*B*_ and *ET*_*B*→*L*_ (*for *p* < 0.005, **for *p* < 0.001, ***for *p* < 0.0001). We used Wilcoxon rank-sum test to calculate the significance^34^.

We observed a statistically significant difference between *ET*_*L*→*B*_ and *ET*_*B*→*L*_ when 10 ≤ Δ*t* ≤ 110. This is because the directional effect from language to biological evolution was weak relative to that from biological to language evolution when we took measurements at short time scales. We think that this is due to the high evolutionary speed of language in short time scales. When we measured the evolutionary rate of language in short time scales, the rate tended to be high because of the measurement-interval dependence described in the Introduction. Biological evolution seems to be unable to keep pace with language evolution because of such high-speed changes in language at short time scales, which is the “moving target” problem^18^. However, at long time scales (Δ*t* > 110), we found that the difference between them tended to be small. This implies that the directional effects on each other are comparable. This means that at long time scale, biological evolution can track language evolution, and coevolution occurs thanks to both phenotypic plasticity and a constraint imposed on dynamics of language evolution by language ability of agents.

## Conclusion

Investigating the evolutionary changes of language directly is difficult; as often stated, “language does not fossilize”. Therefore, researchers have relied on inferring the evolution of linguistic ability on the basis of fossil remains of human ancestors or analyzing cultural evolution (e.g., the evolution of vocabulary), which has been observed over a short time scale. We believe that further investigating the cultural and biological evolutions of language in a comprehensive manner requires “emergent computational thought experiments”^35^ and “opaque thought experiments” as an alternative methodology, where the consequences follow from the premises in such a non-obvious manner that the consequences can be understood only through systematic enquiry^36^).

From this viewpoint, in this paper we proposed an integrated framework for investigating genetic and cultural language evolution. Based on this framework, we first constructed an agent-based model that captures both the cultural evolution of languages and biological evolution of linguistic faculties, which are expressed in a one-dimensional linguistic space. Second, we analyzed the evolutionary rates of cultural evolution and biological evolution by using our simulation results. Finally, we analyzed the directional effects between cultural evolution and biological evolution by using the transfer entropy calculated from our simulation results.

Our evolutionary experiments showed that, after an initial rapid increase in the number of languages, a cyclic coevolutionary process occurs in which biological evolution and cultural evolution proceed alternately. We observed the genetic assimilation of language into the innate linguistic ability. Eventually, the population reaches languages with high expressiveness. Thompson *et al*. constructed several coevolutionary models for biological evolution of innate cognitive biases on language acquisition and cultural learning of languages based on Bayesian inference^37^. They showed that culture facilitates rapid biological adaptation yet rules out nativism. In other words, behavioral universals arise that are underpinned by weak biases rather than strong innate constraints. However, it should be noted that such reduced selection pressures brought about the genetic diversity in the innate genotypes (Fig. 5 (2)), which bootstrapped further evolution processes (Fig. 5 (3)). Repeated or continuous interactions of biological and cultural evolution processes have also been pointed out by using agent-based models of vowel repertoires^38^.

The analysis of the evolutionary rate showed that the cultural rate of evolution is typically faster than the biological rate; hence, the biological evolution cannot maintain the pace with the cultural evolution. However, biological evolution may become faster as a result of a coevolutionary process, and cultural evolution tends to fluctuate more than biological evolution on a short time scale. The analysis of the directional effects showed that biological evolution appears to be unable to maintain the pace with language evolution on short time scales, while the mutual directional effects are comparable on long time scales. This indicates that language and biological evolution can coevolve. We believe that we must observe their evolutions over longer time scales.

These results partly support Számadó *et al*.’s claims, especially with regard to the way phenotypic plasticity promotes adaptation. In addition to Számadó *et al*.’s claims, we obtained the following insights from our simulation: (1) Diversity across language groups increases the fitness variance, which accelerates the rate of biological evolution. (2) The rate of cultural evolution tends to be restricted by the plasticity of individuals because languages cannot survive outside the linguistic plasticity range of individuals. (3) The rate of cultural change can be slow, especially when individuals reduce their learning cost as they cluster around existing languages with sufficient expressiveness for communication. In contrast to situations with no linguistic conventions among speakers, this tends to cause language evolution to stagnate. We think that the rate of cultural change may be faster when there are no linguistic conventions among speakers and slower when some shared conventions exist among them.
